# New Insights Into Culturable and Unculturable Bacteria Across the Life History of Medicinal Maggots *Lucilia sericata* (Meigen) (Diptera: Calliphoridae)

**DOI:** 10.3389/fmicb.2020.00505

**Published:** 2020-04-08

**Authors:** Naseh Maleki-Ravasan, Nahid Ahmadi, Zahra Soroushzadeh, Abbas Ali Raz, Sedigheh Zakeri, Navid Dinparast Djadid

**Affiliations:** ^1^Malaria and Vector Research Group, Biotechnology Research Center, Pasteur Institute of Iran, Tehran, Iran; ^2^Department of Parasitology, Pasteur Institute of Iran, Tehran, Iran; ^3^Department of Biotechnology, Faculty of Advanced Sciences and Technology, Pharmaceutical Sciences Branch, Islamic Azad University, Tehran, Iran

**Keywords:** resident bacteria, blowflies, maggot debridement therapy, forensic entomology, metagenetics, *16S rRNA*

## Abstract

Because of the nutritional ecology of dung- and carrion-feeding, bacteria are the integral part of *Lucilia sericata* life cycle. Nevertheless, the disinfected larvae of the blowfly are applied to treat human chronic wounds in a biosurgery named maggot debridement therapy (MDT). To realize the effects of location/diet on the gut bacteria, to infer the role of bacteria in the blowfly ecology plus in the MDT process, and to disclose bacteria circulating horizontally in and vertically between generations, bacterial communities associated with *L. sericata* specimens from various sources were investigated using culture-based and culture-independent methods. In total, 265 bacteria, including 20 families, 28 genera, and 40 species, were identified in many sources of the *L. sericata*. Culture-dependent method identified a number of 144 bacterial isolates, including 21 species, in flies reared in an insectary; specimens were collected from the field, and third-instar larvae retrieved from chronic wounds of patients. Metagenetic approach exposed the occurrences of 121 operational taxonomic units comprising of 32 bacterial species from immature and adult stages of *L. sericata*. Gammaproteobacteria was distinguished as the dominant class of bacteria by both methods. Bacteria came into the life cycle of *L. sericata* over the foods and transovarially infected eggs. *Enterococcus faecalis*, *Myroides phaeus*, *Proteus* species, *Providencia vermicola*, and *Serratia marcescens* were exchanged among individuals via transstadial transmission. Factors, including diets, feeding status, identification tool, gut compartment, and life stage, governed the bacteria species. Herein, we reemphasized that *L. sericata* is thoroughly connected to the bacteria both in numerous gut compartments and in different life stages. Among all, transstadially transmitted bacteria are underlined, indicating the lack of antagonistic effect of the larval excretions/secretions on these resident bacteria. While the culture-dependent method generated useful data on the viable aerobic gut bacteria, metagenomic method enabled us to identify bacteria directly from the tissues without any need for cultivation and to facilitate the identification of anaerobic and unculturable bacteria. These findings are planned to pave the way for further research to determine the role of each bacterial species/strain in the insect ecology, as well as in antimicrobial, antibiofilm, anti-inflammatory, and wound healing activities.

## Introduction

Insects are known as multiorganismal animals because they are colonized by numerous microorganisms, especially bacteria, in the intestinal tract. This compartment provides a particular setting for microbial colonization where its inhabitants have easy access to food-related microbes, can consume copious amounts of nutrients, and can be protected against the external disturbances (Douglas, 2015). The microbiota adapting to such environment, the resident bacteria, are quite different from transient ones found in the surrounding environment (Engel and Moran, 2013). These beneficial bacteria can typically improve host fitness through contributing to nutrition, reproduction, tolerance to environmental perturbations, maintenance and/or enhancement of host immune system homeostasis, mucosal barrier fortification, colonization resistance, xenobiotic metabolism, ecological communication, defense, speciation, and pathogen transmission ability (O’Neill et al., 1997; Dillon and Dillon, 2004; Ma et al., 2012; Engel and Moran, 2013; Douglas, 2015; Andongma et al., 2018; Maleki-Ravasan et al., 2015, 2019).

The gut microbiota of insects is affected by both what they consume and where they exist. *Lucilia sericata* (Meigen) (Diptera: Calliphordidae), a synanthropic blowfly, frequently feeds and breeds on the carrion, open wounds, feces, and garbage to supply large quantities of proteins required for the development of the progeny (Tomberlin et al., 2011; Pezzi et al., 2015; Junqueira et al., 2017). As a holometabolous insect, *L. sericata* represents four developmental stages in their life cycle, including eggs, three larval instars (L_1_–L_3_), pupae, and adults (Pruna et al., 2019). After mating, the adult females lay cluster of ∼200 eggs at a time on decomposing materials (Zumpt, 1965). The microbial volatile organic compounds (MVOCs), as well as semiochemicals from feeding con- and hetero-specific females, are the key modulators of the fly behavior in the attraction or repulsion of the feeding/breeding resources (Ma et al., 2012; Brodie et al., 2015b). The blowflies can potentially modify microbial communities of the breeding matrices to the beneficial ones through both antimicrobial actions of the larvae and residing symbiotic microbiota (Erdmann and Khalil, 1986; Sherman et al., 2000; Čeřovský et al., 2010; Singh et al., 2015). The anatomy of larvae is optimized in such way to uptake large amounts of foods, that is, 25 mg per larvae within 24 h (Mumcuoglu, 2001). Their simple body structure consists of a pair of salivary glands, a very flexible crop, a tripartite gut (foregut, midgut, and hindgut), four Malpighian tubules, liver-like fat body, a simple central nerve ganglion, and tracheal tubes delivering oxygen directly (Mumcuoglu et al., 2001; Boonsriwong et al., 2011; Baumann, 2017). As a consequence of extracorporeal digestion, larvae secrete a plentiful of digestive enzymes (mainly from the salivary glands) into the substrate to predigest the tissue, which is subsequently swallowed back (Andersen et al., 2010; Wilson et al., 2016). The food and bacteria are degraded enzymatically in the alimentary channel, especially in the midgut lacking chitin and glycoproteins (Terra and Ferreira, 1994; Lerch et al., 2003). In this compartment, a physical barrier posed by the peritrophic matrix separates the food bolus from the midgut epithelium, to prevent the abrasion of midgut (Douglas, 2015) and to inhibit bacterial colonization. The engorged bacteria are removed at this point, and nutrients are absorbed into the hemolymph (Andersen et al., 2010). The drainage pipes, Malpighian tubules, sieve excretion products from the hemolymph and combine them with digested food coming from the midgut (Baumann et al., 2017). The final breakdown of excretion products and nutrition uptake take place in the hindgut, and waste materials are excreted over the anus. Accordingly, during the digestion process, the number of bacteria ingested by larvae reduces due to mechanical, enzymatic, and symbiotic activities throughout the digestive tract (Mumcuoglu et al., 2001; Valachova et al., 2014b).

Because of aforesaid nutritional ecology, *L. sericata* is at the forefront of applied biological sciences. The immature stages are ripened within the decomposing carrion or animal remains, which their developmental data can be exploited for the estimation of the time elapsed since death, denoted as postmortem interval (PMI) in the forensic entomology (Cervantès et al., 2018). Likewise, it is one of the facultative parasites of animals and humans causing wound myiasis worldwide (Francesconi and Lupi, 2012). The controlled therapeutic usage of this kind of myiasis is termed maggot debridement therapy (MDT) (Sherman, 2009). Maggot debridement therapy is currently addressed as biosurgery in which live blowfly larvae are used to cure chronic wounds persistently infected with drug-resistant bacteria (Wollina et al., 2000; Cowan et al., 2013; Bazalinski et al., 2019). The outline of modern MDT was based on the clinical trials conducted in 1990s (Sherman et al., 1995; Fleischmann et al., 2004). Larvae contribute to wound healing process, namely, debridement, disinfection, and regeneration of tissues through the physical contact and release of either fecal waste excretions or salivary gland secretions (ES) containing antimicrobials (Sherman, 2014; Valachova et al., 2014b).

The removal of both necrotic tissue and pathogenic bacteria, called debridement, is a crucial and the best studied stage in the MDT (Sherman, 2014). Larvae debride the wound beds through mechanical activities of mouth hooks or the extracorporeal digestion mediated by proteases and nucleases in the ES (Andersen et al., 2010; Valachova et al., 2014a; Teh et al., 2017). Until now, the occurrence of three classes of proteolytic enzymes, comprising serine proteases, aspartic proteases, and metalloproteases, has been revealed in the maggot ES (Chambers et al., 2003; Alipour et al., 2017). Furthermore, tissue-specific expression of some of these proteases has been determined in the larval body (Franta et al., 2016).

In the disinfection phase, a variety of antimicrobial peptides (AMPs) and small molecules are released to clean up the wound environment (Bexfield et al., 2008; Pöppel et al., 2015). These compounds might be produced either by larvae (Valachova et al., 2014a) or by residing symbionts. A number of 47 AMP genes are encoded by *L. sericata*, representing the highest amount of AMPs among dipteran species (Pöppel et al., 2015). The production of two antibacterial substances, phenylacetic acid and phenylacetaldehyde, in the blowfly larva of *Cochliomyia hominivorax* was attributed to a symbiotic bacterium, *Proteus mirabilis* (Erdmann and Khalil, 1986). In addition, pathogenic microbes are actively picked up by maggots and destroyed within the digestive tract (Mumcuoglu et al., 2001; Valachova et al., 2014b).

The larval ES has exhibited antimicrobial activity against both Gram-positive and Gram-negative bacteria (Bexfield et al., 2008; Yan et al., 2018), as well as against protozoan parasites causing dermal leishmaniasis (Polat et al., 2012; Sanei-Dehkordi et al., 2016). Thus, suppressing the pathogenic microbes on the feeding substrates promotes the larval development and survival (Sherman et al., 2000). Maggots can manage this function via selective antimicrobial activity against pathogenic and symbiotic bacteria (Jaklic et al., 2008). It has been suggested that infected environments could influence the larval antibacterial activity; thus, infected larvae show stronger antibacterial capacities than germ-free larvae (Kawabata et al., 2010). Furthermore, maggot proteases have been displayed to be up-regulated upon immune challenges (Altincicek and Vilcinskas, 2009) and responsible for the inhibition or degradation of bacterial biofilm (Harris et al., 2013).

Maggots induce tissue growth via mechanical and biochemical stimulation of healthy cells. They induce the release of host growth factors and reduce debris and biofilm or microbial loads, which likely decreases inflammation and promotes wound healing (Sherman, 2014). It is thought that the alkalinity of maggot-treated wounds, together with the isolation of urea-containing compounds, for example, allantoin, is responsible for wound healing stimulation (Robinson, 1935; Baumann et al., 2017). Other basic mechanisms of wound healing, including inhibition of complement activation, down-regulation of the C3a/C5a-mediated neutrophil activation, and regulation of MMP-2 and MMP-9 expressions modulated by AP-1 (c-jun), have been taken into account in recent years (Tamura et al., 2017; Tombulturk et al., 2019).

According to the established background, bacteria, especially resident ones, are an integral part of *L. sericata* life cycle and presumably play an important role in the wound healing process. Diets and breeding environments will also have a great influence on the blowfly gut bacterial profile. Flies are reciprocally dependent on specific bacteria and their metabolic pathways for the growth and development (Zurek et al., 2000; Crooks et al., 2016). Most studies have shown that the best survival rates of flies occur in the unsterilized or mixed bacterial environments (Tomberlin et al., 2017). These considerations that raise several noteworthy inquiries about the potential bacterial groups associated with these biosurgeon flies need to be addressed in detail. To realize the effects of diets and rearing matrices on the gut bacteria, to infer the role of the gut bacteria in the blowfly ecology and to disclose bacteria transmitted horizontally in and vertically between generations, this descriptive cross-sectional study was designed to survey the bacterial communities of *L. sericata* specimens from different resources.

## Materials and Methods

### Blowfly Colony

For mass rearing, a colony of *L. sericata* (Garmdarreh strain) was established in the National Insectary of Iran (NII, MVRG), located at the Production and Research Complex of Pasteur Institute of Iran (Karaj), from summer 2009. The adult flies were kept in cages of 50 × 50 × 50 cm at 22.5°C ± 5°C mean temperature, 35 ± 10% relative humidity, and 12 h photoperiodicity. They were provided with cotton balls saturated with 10% sugar solution. New individuals had never been added from field to the colony, but extra-generation breeding was carried out repeatedly. Oviposition substrates, consisting of chicken liver plus sawdust, were introduced to the mated females when needed to extend generations. The eggs were transferred to the maggotarium to continue the life cycle of the blowfly or were externally disinfected to apply in the MDT. Flies from the 400th generation were used for microbiological surveys.

### External Disinfection of Blowfly Eggs

To decrease the bacterial load on the *L. sericata* eggs, Lysol immersion method was applied as described by Brundage et al. (2016). Briefly, freshly deposited eggs (16–18 h old) were immerged in 3% Lysol for 10 min and then rinsed in 10 cc of 70% EtOH and 30 cc of 1% NaOCl. The sterilized eggs were transferred to the blood agar medium and incubated aerobically at 37°C overnight. A number of larvae were allowed to develop in this sterile medium to check their gut bacterial flora.

### Culture-Dependent Identification of Bacteria

#### Sampling

To investigate cultivable gut bacteria, we used three types of fly specimens: samples from NII collections, newly collected samples from the field, and samples retrieved from the MDT of patients.

A total of 26 specimens were selected from the NII to investigate their microbiota. The specimens included two larval and adult food supplies, six developmental stages of eggs, L_1_ and L_2_ rearing on the sterile/non-sterile diets, 13 microdissected compartments (Supplementary Figure S1) from the digestive tract of L_3_ (salivary glands, crop, foregut, midgut, hindgut, and Malpighian tubules, before/after feeding plus the trachea of respiratory tract), and five specimens from mature stages (pupae and male/female adults that their excreta and adult corpse were preserved for the long term). All the samples were processed in triplicates.

Field samples were collected from Garmdarreh City (35°45′49″ N, 51°3′44″ E). Adult flies were gathered using chicken-liver baited traps. Captured flies were transferred to the NII and morphologically identified. Six adult blowflies (including three *L. sericata* and three *Calliphora* species) were dissected for bacteriological assay, and the rest were kept for progeny production. Of the new generation, six samples, including three L_3_ and three pupae, were examined as well.

Two bed sore and diabetic foot patients (Supplementary Figures S2, S3), who were under MDT, were screened to find which bacteria are consumed by the maggots. The origin of the larvae used for the MDT of these patients was from the NII. 3 days after MDT, the third-stage larvae were collected from the wounds, and nine specimens were examined bacteriologically.

#### Sample Preparation

Prior to dissection, the flies were killed at −20°C for 3 min. The specimens were rinsed twice with phosphate-buffered saline (PBS) to remove the attached particles. Subsequently, to surface sterilize, they were immerged twice in 70% ethanol for 2 min. All the specimens were then dissected aseptically within a drop of sterile PBS on a sterile glass slide, under a laminar flow hood. Each dissected compartment (or whole bodies of eggs and pupae) was homogenized in 100 μL of PBS. The solution was entirely inoculated into the brain heart infusion (BHI) broth medium.

#### Bacteriological Methods

Several enrichment and selective culture media, comprising of BHI broth, BHI agar, MacConkey agar, and phenylethyl alcohol agar (PEA), were used to cultivate bacteria aerobically at 37°C overnight. Following the initial selection of the BHI broth medium, the positive samples were subcultured in the medium. To obtain individual pure colonies, the grown bacteria were serially diluted or streaked on specific media (MacConkey agar for Gram-negative bacteria and PEA for Gram-positive bacteria). Furthermore, to prevent bacterial swarming (rapid and coordinated translocation of bacteria, e.g., *Proteus* species), which arrests the growth of other bacteria and the achievement of pure individual colonies, methods such as agar-enriched BHI broth medium, PEA, and pour plate were employed.

#### Molecular Identification of Pure Colonies

The genomic DNA of bacteria was prepared using a commercial kit (Molecular Biological System Transfer [MBST], Tehran, Iran), according to the manufacturer’s instructions. The universal primers of 16suF: 5′-GAGTTTGATCCTGGCTCAG-3′ and 16suR: 5′-GTTACCTTGTTACGACTT-3′ were used to amplify ∼1,450 bp of the *16S rRNA* gene. The amplification and sequencing were carried out based on the methods described previously (Weisburg et al., 1991; Maleki-Ravasan et al., 2015). Using a UV transilluminator, polymerase chain reaction (PCR) products were visualized on a 1% agarose gel stained with GreenView^TM^, Parstous Biotechnology, Mahhad, Iran. Amplicons were separated from the gel, and after purification, they were sequenced bidirectionally using the same amplification primers by the Macrogen Company, Tehran, Iran.

A biochemical test using EMB agar (eosin methylene blue agar) medium was also applied for the identification of the *Escherichia coli* and *Shigella* species, which share identical *16S rRNA* sequences, as described by Leininger et al. (2001; Supplementary Figure S4).

#### Antimicrobial Susceptibility of *P. mirabilis* Isolates

Using disk diffusion method, the *P. mirabilis* isolates were subjected to antibiotic susceptibility testing against 22 antibiotics representing eight families. The origin of bacteria were from six different microdissected compartments of L_3_ including salivary glands (*n* = 4), foregut (*n* = 4), midgut (*n* = 2), hindgut (*n* = 2), Malpighian tubules (*n* = 1), and trachea (*n* = 1). The antibiotics examined against the isolates included amikacin, azithromycin, bacitracin, ceftazidime, chloramphenicol, ciprofloxacin, colistin, cefotaxime, erythromycin, imipenem, kanamycin, meropenem, novobiocin, neomycin, optochin, penicillin, piperacillin, ampicillin + sulbactam, streptomycin, tetracycline, trimethoprim, and vancomycin. Inhibition zone diameter of each antimicrobial disc was measured, and the isolates were categorized as resistant, intermediate, and susceptible.

### Culture-Independent Identification of Bacteria

#### Sampling

A total of 36 specimens were used in the amplicon-based metagenomic survey of bacteria circulating in the life cycle of *L. sericata*. The specimens included three of each developmental stage of eggs, the L_1_ and L_2_, pupae, and male and female adults, as well as three of each microdissected compartment of the digestive tract of L_3_, including salivary glands, crop, foregut, midgut, hindgut, and Malpighian tubules. All the specimens were originated from the NII. The conditions for sample preparation, including anesthetizing, surface sterilizing, and dissection, were the same as mentioned before. The total DNA of each dissected tissue was directly subjected to the bacterial identification.

#### DNA Extraction, Primer Design, and PCR

Total genomic DNA of individual tissues was extracted to identify intercellular/intracellular bacteria using “tissue protocol” of MBST kit, following the manufacturer’s guidelines. A nested PCR assay was conducted to raise the sensitivity of PCR assay in direct detection of bacteria from the insect tissues. In the first step of the nested PCR, the universal primers 16suF and 16suR were used to amplify the whole of nine hypervariable regions (V1–V9) in the bacterial *16S rRNA* genes. In the second step, a large number of *16S rRNA* gene sequences belonged to bacterial families, including clinical to environmental species, were subjected to primer designing based on the V1–V5 regions. Two universal primers, Nest2F (5′-GCRKGCCTAAYACATGCAAG-3′) and Nest2R (5′-CGTGGACTACCAGGGTATCTAATC-3′), were designed to amplify ∼800 bp of the gene. The PCR product of the first stage was used as a template DNA for the next step. The PCR reaction was performed using 50 ng of PCR product, 10 picomoles of the primers, 1 mM of dNTP, 1 U of *Taq DNA* polymerase (CinnaGen Company, Tehran, Iran), and PCR buffer. Polymerase chain reaction conditions included an initial denaturation step of 94°C for 5 min, followed by 10 cycles of 94°C for 30 s, 58°C for 30 s, and 72°C for 90 s, and 25 cycles of 94°C for 30 s, 57°C for 30 s, and 72°C for 90 s, which was accompanied by a final extension at 72°C for 10 min. For each sample, the second stage of nested PCR reaction was repeated four times in a total volume of 20 μ L.

### Cloning and Sequencing

The second-stage PCR products were separated on a 1.5% agarose gel and then purified using GF-1 PCR Clean-up Kit; Vivantis, Shah Alam, Selangor Darul Ehsan, Malaysia. The inserts (∼800-bp-long PCR products) were ligated into the PGEM-T EASY Vector (Promega, Madison, Wisconsin, United States) using T4 DNA ligase (Fermentas, Waltham, Massachusetts, United States). This complex was transformed into DH5α strain of *E. coli*. The positive colonies (20–30 clones) were checked for the presence of inserts through the approaches of colony PCR and digestion with *Eco*RI. The plasmid DNA was extracted from the insert-positive colonies using the GF-1 Plasmid DNA Extraction Kit (Vivantis) and commercially sequenced with a Sanger platform by using the M13F and M13R vector primers at Macrogen Company.

### Data Analysis

All successful *16S rRNA* sequences were analyzed to assign the correct scientific name of bacterial species. The last version of software DECIPHER (Wright et al., 2012) was used to check the probable chimeric sequences within *16S rRNA* gene clone library, and the specimens with suspicious sequences were removed from the data. The consensus of confident sequences was therefore analyzed using databases available for *16S rRNA* genes of prokaryotes, including NCBI (*16S rRNA* sequences), EzBioCloud, and leBIBI (Supplementary Tables S1, S2). The MEGA5 software was utilized for the comparative analysis of the sequences and phylogenetic tree construction. Position verifications and phylogenetic inference were conducted using maximum likelihood method with 1,000 bootstrap replicates. The sequences data were deposited in the GenBank database. Venn diagram of all classified sequences was created using the software VENNTURE (Martin et al., 2012).

## Results

### General Overview of Identified Bacteria

**FIGURE 1:**
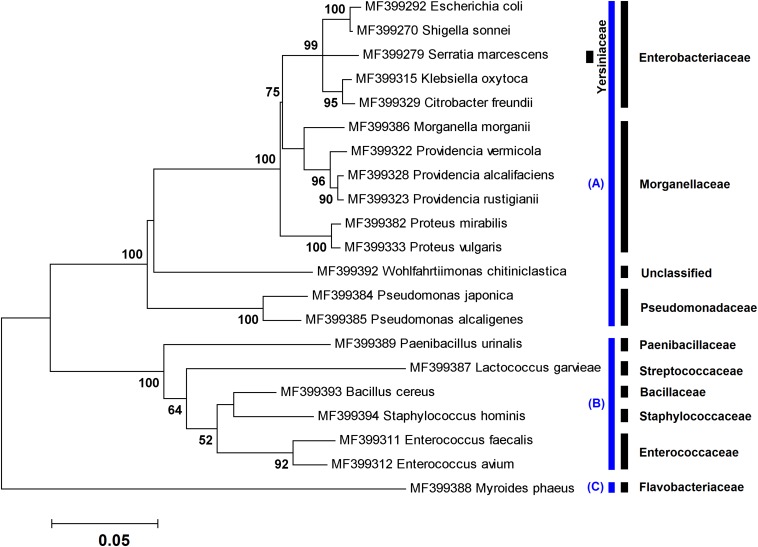
Maximum likelihood tree showing the phylogenetic relationships of ∼1,400 bp of the *16S rRNA* gene sequences of 21 species obtained in this study using culture-dependent method. The numbers at the branch points are bootstrap values based on 1,000 replicates. The cutoff values lower than 50% are not shown. All species were classified into three phyla: **(A)** Proteobacteria, **(B)** Firmicutes, and **(C)** Bacteroidetes. The corresponding family taxa are indicated in the front of branches.

**FIGURE 2:**
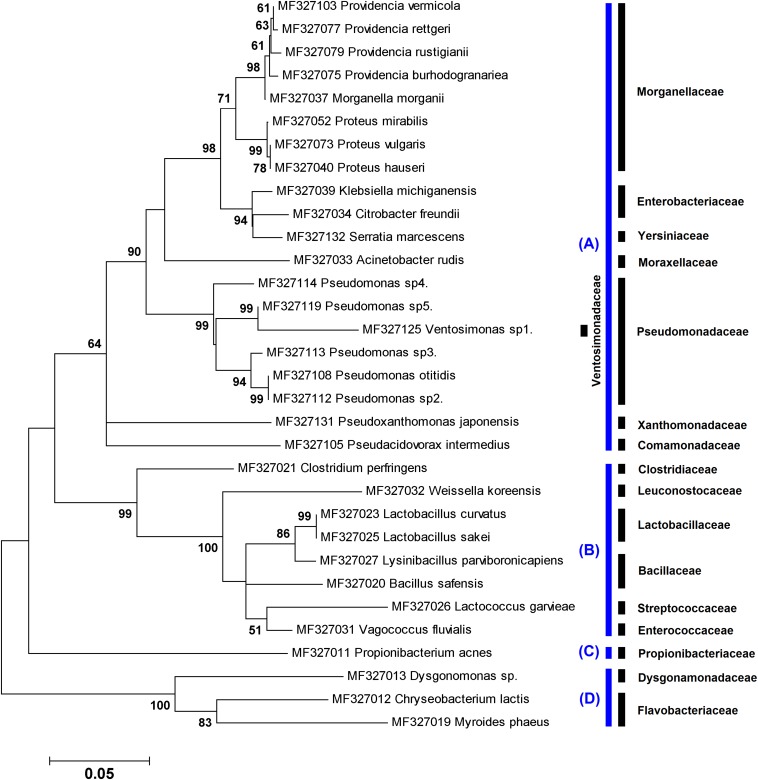
Maximum likelihood tree showing the phylogenetic relationships of ∼800 bp of the *16S rRNA* gene sequences of 32 species obtained in this study using culture-independent method. The numbers at the branch points are bootstrap values based on 1,000 replicates. The cutoff values lower than 50% are not shown. All species were classified into three phyla: **(A)** Proteobacteria, **(B)** Firmicutes, **(C)** Actinobacteria, and **(D)** Bacteroidetes. The corresponding family taxa are indicated in the front of branches.

**FIGURE 3:**
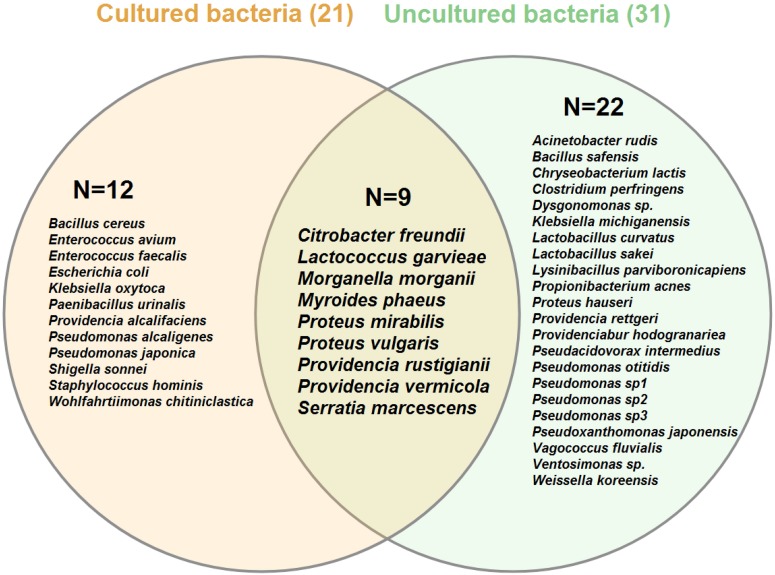
Venn diagram of bacterial species associated with different life stages of *Lucilia sericata* arranged by the isolation method: culture-dependent (cream circle) versus culture-independent (light green circle). Numbers in parentheses indicate the total number of species identified by each method. Numbers inside the circles show unique/shared bacteria recognized by two identification methods. Venn diagram was created using VENNTURE program (Martin et al., 2012).

**FIGURE 4:**
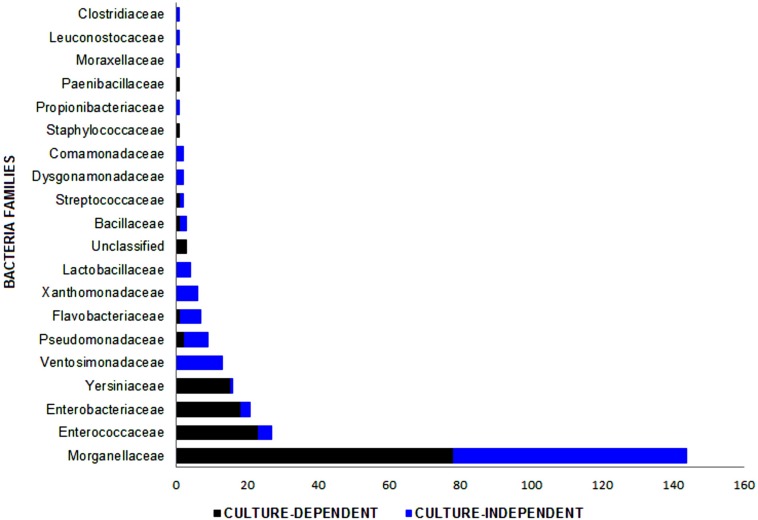
Bacterial families identified in the *Lucilia sericata* life cycle by two culture-dependent and culture-independent methods. Unclassified family includes three strains of *Wohlfahrtiimonas chitiniclastica*.

In total, 265 bacterial isolates, including 20 families, 28 genera, and 40 species, were identified from different sources of the *L. sericata* specimens (Tables 1–3). The isolates were belonging to four phyla, including Proteobacteria (81.13%), Firmicutes (15.09%), Bacteroidetes (3.40%), and Actinobacteria (0.38%) (Figures 1, 2 and Supplementary Tables S1, S2). The number of 21 and 32 unique species was recognized by two culture-dependent and metagenetic methods, respectively. Nine species were identified by both methods, as well (Figure 3). Morganellaceae and *Proteus* species were the most abundant identified family and genus of bacteria, correspondingly (Figure 4 and Tables 1–3). The consensus sequences were deposited in the GenBank under accession numbers MF399269-MF399394 for cultured and MF327011-MF327133 for uncultured bacteria.

**TABLE 1:** Details of bacteria found in the *Lucilia sericata* life cycle reared in the National Insectary of Iran (NII).

Isolation source/Bacteria species	Food sources		Immature stages										Mature stages					No.
	Sugar meal	Chicken liver	Reared on the sterile/non-sterile diet			Microdissected third-stage larvae							Pupae	Male	Female	Adults excreta	Corpse of adult flies	
			Egg	L_1_	L_2_	SG	Cr	FG	MG	HG	MT	Tr						
*Acinetobacter rudis*	–	–	–	–	–	–	–	–	–	–	–	–	–	(1)	–	–	–	1
*Bacillus safensis*	–	–	–	–	–	–	–	–	–	–	–	–	–	(1)	–	–	–	1
*Chryseobacterium lactis*	–	–	–	–	–	–	–	–	–	–	(1)	–	–	–	–	–	–	1
*Citrobacter freundii*	–	–	[1](1)	–	–	–	–	–	–	–	–	–	–	–	–	–	–	2
*Clostridium perfringens*	–	–	(1)	–	–	–	–	–	–	–	–	–	–	–	–	–	–	1
*Dysgonomonas* species	–	–	–	–	–	–	–	–	–	–	–	–	–	–	(2)	–	–	2
*Enterococcus faecalis*	–	1	–	[2]	–	–	–	{1}	–	–	–	–	2	1	1	1	–	9
*Escherichia coli*	–	1	[1]	–	–	–	–	–	–	–	–	–	–	–	–	3	–	5
*Klebsiella michiganensis*	–	–	–	(1)	–	–	–	(1)	–	–	–	–	–	–	–	–	–	2
*Klebsiella oxytoca*	1	–	–	1	–	–	–	–	–	–	–	–	–	–	2	–	–	4
*Lactobacillus curvatus*	–	–	(1)	(1)	–	–	–	–	–	–	–	–	–	–	–	–	–	2
*Lactobacillus sakei*	–	–	–	(1)	(1)	–	–	–	–	–	–	–	–	–	–	–	–	2
*Lactococcus garvieae*	–	–	–	[1]	–	(1)	–	–	–	–	–	–	–	–	–	–	–	2
*Lysinibacillus parviboronicapiens*	–	–	(1)	–	–	–	–	–	–	–	–	–	–	–	–	–	–	1
*Morganella morganii*	–	–	–	(2)	–	–	–	–	{1}	–	–	–	(1)	–	–	–	–	4
*Myroides phaeus*	–	–	(2)	–	(1)	–	1	–	–	–	(1)	–	–	1	–	–	–	6
*Paenibacillus urinalis*	–	–	–	–	–	–	{1}	–	–	–	–	–	–	–	–	–	–	1
*Propionibacterium acnes*	–	–	–	–	–	–	–	–	–	–	–	–	–	(1)	–	–	–	1
*Proteus hauseri*	–	–	–	–	–	–	–	–	–	–	–	–	(1)	–	–	–	–	1
*Proteus mirabilis*	–	–	[1]	[1]	[3](2)2	{3}1(1)	{4}(1)	{1}1	{2}1(2)	{4}1(2)	{2}2(3)	4	3	(4)	4	1	–	56
*Proteus vulgaris*	–	–	[1]	[1]	[1](1)	–	–	(17)	–	–	–	–	–	–	–	1	–	22
*Providencia alcalifaciens*	–	–	3	–	–	–	–	–	–	–	–	–	–	1	–	–	–	4
*Providencia burhodogranariea*	–	–	–	–	(1)	–	–	–	–	–	–	–	–	–	–	–	–	1
*Providencia rettgeri*	–	–	–	–	–	–	–	–	(1)	(1)	–	–	–	–	–	–	–	2
*Providencia rustigianii*	–	–	–	[2]	(2)	–	–	–	–	–	–	–	–	–	–	–	–	4
*Providencia vermicola*	–	–	–	–	2(5)	{1}	–	–	(3)	–	(1)	–	–	1(15)	–	–	–	28
*Pseudacidovorax intermedius*	–	–	–	–	–	–	–	(1)	–	–	–	–	–	(1)	–	–	–	2
*Pseudomonas alcaligenes*	–	–	–	–	–	{1}	–	–	–	–	–	–	–	–	–	–	–	1
*Pseudomonas japonica*	1	–	–	–	–	–	–	–	–	–	–	–	–	–	–	–	–	1
*Pseudomonas otitidis*	–	–	(1)	–	–	–	–	(3)	–	–	–	–	–	–	–	–	–	4
*Pseudomonas* sp3	–	–	–	–	(1)	–	–	–	–	–	–	–	–	–	–	–	–	1
*Pseudomonas* sp4	–	–	–	–	–	–	–	–	–	–	(1)	–	–	–	–	–	–	1
*Pseudomonas* sp5	–	–	–	–	–	–	–	–	–	–	–	–	–	–	(1)	–	–	1
*Pseudoxanthomonas japonensis*	–	–	–	–	(1)	–	–	(4)	–	–	–	–	–	(1)	–	–	–	6
*Serratia marcescens*	2	–	–	2	[1]2	{1}	–	–	{1}	1	{1}(1)	–	–	–	–	–	4	16
*Shigella sonnei*	–	2	–	[1]	–	–	–	–	–	–	–	–	–	–	–	–	–	3
*Staphylococcus hominis*	–	–	–	–	–	–	–	–	–	–	–	–	1	–	–	–	–	1
*Vagococcus fluvialis*	–	–	(1)	–	(2)	–	–	–	–	–	(1)	–	–	–	–	–	–	4
*Ventosimonas sp1*	–	–	–	(3)	–	–	–	–	–	–	–	–	–	–	(10)	–	–	13
*Weissella koreensis*	–	–	(1)	–	–	–	–	–	–	–	–	–	–	–	–	–	–	1
Total	4	4	16	19	28	9	7	29	11	9	14	4	8	28	20	6	4	220

*Numbers in braces, brackets, and parentheses represent bacteria detected in the unfed state of the digestive tract, bacteria identified from specimens reared on the sterile diet, and bacteria identified by metagenetic approach, respectively. L_1_–L_3_, first to third larval stages; SG, salivary glands; Cr, crop; FG, foregut; MG, midgut; HG, hindgut; MT, Malpighian tubules; Tr, trachea.*

**TABLE 2:** Bacteria found in trapped blowflies in the field.

Isolation source/Bacteria species	*Lucilia sericata*				*Calliphora* species				No.
	L_3_	Pupae	Male	Female	L_3_	Pupae	Male	Female	
*Enterococcus faecalis*	1	−	−	−	−	−	−	2	3
*Proteus mirabilis*	3	1	−	−	4	−	−	2	10
*Providencia vermicola*	1	−	1	−	−	1	−	−	3
*Escherichia coli*	−	1	−	−	−	−	−	−	1
Total	5	2	1	−	4	1	−	4	17

**TABLE 3:** Bacteria found in the third-stage larvae retrieved from two bed sore and diabetic foot patients.

Isolation source/Bacteria species	Patient with bed sores	Patient with diabetic foot	No.
*Proteus mirabilis*	8	−	8
*Enterococcus faecalis*	7	3	10
*Bacillus cereus*	1	−	1
*Wohlfahrtiimonas chitiniclastica*	3	−	3
*Morganella morganii*	−	1	1
*Escherichia coli*	1	3	4
*Enterococcus avium*	1	−	1
Total	21	7	28

### Culture-Dependent Identification of Bacteria

In culture-dependent method, a number of 144 bacterial isolates, including 21 species, were identified in specimens rearing in the NII (*n* = 19), flies collected from the field (*n* = 4), and L_3_ retrieved from two patients (*n* = 7) (Tables 1–3). Phylogenetic analysis of the cultured bacteria based on ∼1,400 bp of the *16S rRNA* gene sequences showed that the isolates were belonging to three phyla, Proteobacteria (80.56%), Firmicutes (18.75%), and Bacteroidetes (0.69%) (Figure 1).

Bacteria entered the *L. sericata* life cycle through the foods (which may be inoculated by flies) and transovarially infected eggs. Six bacterial species were isolated from larval and adult food supplies. They included *Klebsiella oxytoca*, *Pseudomonas japonica*, and *Serratia marcescens* from sugar meal and *Enterococcus faecalis*, *E. coli*, and *Shigella sonnei* from chicken liver. Results showed the presence of nine and five bacterial species from developmental stages rearing on the sterile and non-sterile diets, respectively (Table 1). The bacteria of *P. mirabilis* and *S. marcescens* were shared between two types of diets. Moreover, larvae reared on a sterile diet generally did not grow up to the L_3_ and if grown, the larvae were very small. Four species, *Citrobacter freundii*, *E. coli*, *P. mirabilis*, and *Proteus vulgaris*, were found in sterilized eggs and only one species, *Providencia alcalifaciens*, in non-sterile eggs. Regardless of whether they were sterilized, a number of eight, four, and nine bacterial species were detected in the L_1_, L_2_, and L_3_, respectively (Table 1).

Three bacterial species, namely, *E. faecalis*, *P. mirabilis*, and *Staphylococcus hominis*, were isolated from pupal stage. Five species, including *E. faecalis*, *E. coli*, *P. mirabilis*, *P. vulgaris*, and *K. oxytoca*, were found in adult males and females; the first four species were detected in fly’s excreta. The bacterium *S. marcescens* was isolated from the corpse of adult flies, which had been preserved in a dry condition for more than 2 years (Table 1). *Proteus* species (*n* = 19) and *E. faecalis* (*n* = 8) were detected in both immature and mature stages of *L. sericata*, indicating the transstadial transmission of these bacteria between the larval stages and adults.

There were nine bacterial species in six compartments of the digestive tract and the trachea of respiratory tract of L_3_ (Table 1). The *P. mirabilis* was the most abundant bacterium in all the studied materials. Bacteria in the salivary glands were more diverse than those observed in other compartments (Table 1). Bacterial flora in the L_3_ was examined both before and after feeding. Seven (*E. faecalis*, *Morganella morganii*, *Paenibacillus urinalis*, *P. mirabilis*, *P. vermicola*, *Pseudomonas alcaligenes*, and *S. marcescens*) and three (*Myroides phaeus*, *P. mirabilis*, and *S. marcescens*) species were detected in the unfed and fed larvae, respectively (Table 1).

Four bacterial species, *E. faecalis*, *E. coli*, *P. mirabilis*, and *P. vermicola*, were identified in trapped blowflies in the field of Garmdarreh City (Table 2). However, 28 bacterial isolates, including seven species, were detected in two bed sore and diabetic foot patients (Table 3). Four out of seven species of the bacteria (*E. faecalis*, *E. coli*, *M. morganii*, and *P. mirabilis*) were also observed in the NII specimens, but only three (*Bacillus cereus*, *Enterococcus avium*, and *Wohlfahrtiimonas chitiniclastica*) were new isolates that were absent in the digestive tract of our previously tested specimens.

### Antimicrobial Susceptibility of *P. mirabilis* Isolates

Antibiogram results showed several *P. mirabilis* isolates with different biochemical properties in the digestive tract of *L. sericata*. Results also revealed that all the 14 studied isolates were resistant to the seven antibiotics, including bacitracin, colistin, erythromycin, streptomycin, tetracycline, trimethoprim, and vancomycin. Susceptibility test findings of five antibiotics, that is, ampicillin/sulbactam, cefotaxime, novobiocin, optochin, and penicillin, were more diverse than other antibiotics and ranged from susceptible and intermediate to resistant. Susceptibility patterns were more noticeable in the salivary glands (*n* = 6) and midgut (*n* = 8) isolates (Table 4).

**TABLE 4:** Antibiogram profile of 14 strains of *Proteus mirabilis* isolated from the six compartments of third-stage larvae of *Lucilia sericata*.

Antibiotic/Compartment	AMK	AZM	BAC	CAZ	CHL	CIP	CST	CTX	ERY	IPM	KAN	MRP	NB	NEO	OPT	PEN	PIP	SAM	STR	TET	TMP	VAN
Foregut	I	S	R	S	R	I	R	S	R	I	R	S	S	I	I	I	S	S	R	R	R	R
	I	S	R	I	R	I	R	S	R	I	R	S	S	I	I	S	S	S	R	R	R	R
	I	S	R	S	R	S	R	S	R	I	R	S	S	I	S	R	S	I	R	R	R	R
	I	S	R	S	R	S	R	S	R	I	R	S	S	I	S	I	S	S	R	R	R	R
Salivary glands	I	S	R	S	R	S	R	S	R	I	R	S	S	I	R	I	S	S	R	R	R	R
	I	S	R	S	R	I	R	S	R	I	R	S	S	I	R	I	S	S	R	R	R	R
	I	S	R	S	R	I	R	S	R	I	R	S	I	I	I	I	S	S	R	R	R	R
	I	S	R	I	R	I	R	S	R	I	R	S	I	I	S	R	S	I	R	R	R	R
Midgut	I	S	R	S	R	I	R	S	R	I	R	S	I	I	I	I	S	S	R	R	R	R
	I	S	R	I	R	I	R	R	R	S	R	I	R	I	I	R	I	R	R	R	R	R
Hindgut	I	S	R	S	R	I	R	S	R	I	R	S	S	I	I	I	S	S	R	R	R	R
	I	S	R	S	R	I	R	S	R	I	R	S	I	I	I	I	S	I	R	R	R	R
Malpighian tubules	I	S	R	S	I	I	R	S	R	I	I	I	I	I	I	R	S	I	R	R	R	R
Tracheae	I	S	R	S	I	I	R	S	R	I	I	S	S	I	I	I	S	I	R	R	R	R

*AMK, amikacin; AZM, azithromycin; BAC, bacitracin; CAZ, ceftazidime; CHL, chloramphenicol; CIP, ciprofloxacin; CST, colistin; CTX, cefotaxime; ERY, erythromycin; IPM, imipenem; KAN, kanamycin; MRP, meropenem; NB, novobiocin; NEO, neomycin; OPT, optochin; PEN, penicillin; PIP, piperacillin; SAM, ampicillin + sulbactam; STR, streptomycin; TET, tetracycline; TMP, trimethoprim; VAN, vancomycin; S, susceptible; I, intermediate; R, resistance.*

### Culture-Independent Identification of Bacteria

A total of 121 chimera-free bacterial operational taxonomic units, including 32 species, were identified in immature (*n* = 26) and adult (*n* = 13) stages of *L. sericata* using metagenetic method. Phylogenetic relationships of the uncultured bacteria, based on ∼800 bp of the *16S rRNA* gene sequences, are illustrated in Figure 2. The bacteria belonged to four phyla that include Proteobacteria (81.82%), Firmicutes (10.74%), Bacteroidetes (6.61%), and Actinobacteria (0.83%).

Eight species of bacteria detected in the eggs were *C. freundii*, *Clostridium perfringens*, *Lactobacillus curvatus*, *Lysinibacillus parviboronicapiens*, *M. phaeus*, *Pseudomonas otitidis*, *Vagococcus fluvialis*, and *Weissella koreensis*. A number of 4, 11, and 15 bacterial species were found in the L_1_ to L_3_ stages, respectively. In microdissected compartments of L_3_ (Supplementary Figure S1), the DNAs of the *Lactococcus garvieae* and *P. mirabilis* were identified in the salivary glands, and only *P. mirabilis* was found in the crop. In addition, six, three, and two bacterial species were detected in the foregut, midgut, and hindgut, respectively. Using metagenetic analysis, most species of bacteria were observed in the Malpighian tubules of L_3_ (*n* = 7). The species *M. morganii* and *Proteus hauseri* were isolated from pupae, whereas eight and three species of bacteria were isolated from male and female adult flies, respectively. The detailed data are listed in Table 1.

## Discussion

This study was designed to investigate bacterial communities associated with the life history of *L. sericata* using culture-dependent and culture-independent methods. In particular, the study investigated bacterial entrance/circulation routes in blowfly, effects of food diets on the gut bacteria, identification of bacteria in different parts of the gut, comparison of bacterial flora of laboratory-reared and field-collected specimens, and bacteria removal by larvae during MDT.

The results of this study reemphasized that *L. sericata* is thoroughly connected to the bacteria because they feed and breed only on organic materials undergoing decomposition processes. We specified the presence of 21 and 32 species of bacteria in *L. sericata* specimens using culture-dependent and metagenetic methods, respectively. Both identification techniques displayed their own pros and cons. While the former generated useful data on the viable aerobic gut bacteria, the latter detected intracellular/extracellular species of bacteria and rapidly identified anaerobic ones with relatively smaller samples. In fact, in the first approach, we lost many anaerobic bacteria (which may be significant), but the second approach covered this defect. The low volume of insect tissues and the length of the *16S rRNA* of the bacteria hindered the detection of bacteria by the conventional *16S rRNA*-PCR; therefore, a pair of primers was developed to utilize in the nested PCR assay. Hence, diverse ranges of bacteria were successfully detected directly from the desired tissues. It is well established that only a small percentage of environmental microbes can be cultivated (Amann et al., 1995). However, metagenetic methods are also varied in the simplicity of extraction of nucleic acids from different bacterial cell types (Head et al., 1998). In total, molecular techniques have raised our knowledge of insect microbiota (Dillon and Dillon, 2004; Engel and Moran, 2013; Yun et al., 2014; Hammer et al., 2015; Singh et al., 2015; Tomberlin et al., 2017).

To explore the insect microbiota, applying both culture-dependent and metagenetic methods would be worthwhile. Results from both methods used in the present investigation indicated that the majority of the identified bacteria (81%) belonged to the Gammaproteobacteria. This result is in agreement with those of a former study in which bacteria associated with different life stages of *L. sericata* and *Lucilia cuprina* were characterized using *16S rDNA* 454 pyrosequencing method (Singh et al., 2015). The aforementioned class of bacteria comprises the laboratory model *E. coli*, human well-known pathogens (e.g., *Salmonella*, *Yersinia*, *Vibrio*, and *Pseudomonas*), and insect endosymbionts (Williams et al., 2010). These bacteria generally display broad ranges of aerobicity, trophism, and temperature adaptation (Scott et al., 2006). As a result, these details should be taken into account when designing studies with the aim of examining the whole microbiota.

In the first part of the study, the BHI broth medium was employed for growing aerobic bacteria because it could promote the growth of nutritionally fastidious and non-fastidious bacteria from a variety of clinical and environmental sources. Nonetheless, we encountered the swarming motility of *Proteus* species that halts the growth of other bacteria on the solid media. This motility was successfully arrested after hardening the BHI agar medium by the elevation of agar concentration (up to 4%). Up to now, ∼400 generations of *L. sericata* have been reared in the NII; hence, the population has become genetically homogeneous. To investigate the effects of this homogeneity and the location on the gut bacteria, the microbiota of field-collected green and blue blowflies was set as a control. Although the number of field collected (uncontrolled conditions) flies was lower than laboratory-reared (controlled conditions) specimens, four identical bacterial species with the dominance of *P. mirabilis* were found in both environments (Tables 1, 2). This evidence could be an emphasis on the presence of native microbiota in the *L. sericata*.

The fact that what kinds of bacteria are removed from a patient’s wound by the larvae during MDT has not been investigated. By identifying L_3_ bacteria removed from patients’ wounds and earlier knowledge on the microbial background of specimens, it can be concluded that *B. cereus*, *E. avium*, and *W. chitiniclastica* have been picked up by larvae from the wounds (Table 3). Generally, *B. cereus* causes serious intestinal or non-intestinal infections through the production of tissue-destructive exoenzymes (Bottone, 2010). *Enterococcus avium*, the most common enterococci in birds, is rarely associated with human bacteremia (Na et al., 2012). *Wohlfahrtiimonas chitiniclastica*, another source of bacteremia, has recently been regarded as an emerging human pathogen (Schröttner et al., 2017). This bacterium may be closely linked to the synanthropic flies, for example, *Wohlfahrtia magnifica*, *L. sericata*, *Chrysomya megacephala*, or *Musca domestica* (Schröttner et al., 2017). Based on our knowledge, this is the first report on the isolation of *W. chitiniclastica* directly from *L. sericata* larvae and indirectly from a 90-year-old female patient with bed sore in Iran.

Literature reviews show that the most common bacterial species associated with both decubitus and diabetic foot infections include *Staphylococcus aureus*, *Staphylococcus epidermidis*, *Staphylococcus lugdunensis*, *P. mirabilis*, *Pseudomonas aeruginosa*, *Enterobacter cloacae*, *E. faecalis*, and *Finegoldia magna* (Dana and Bauman, 2015; Jneid et al., 2018). It is uncertain whether various strains of bacteria such as *P. mirabilis* and *E. faecalis*, which are found in both chronic wounds and larvae used in MDT, are similar, or the strains of a given bacterium distributed in different compartments of the digestive tract of *L. sericata* have the same biochemical properties.

The first query is open and needs to be reflected in detail in future studies. Commensals and pathogens do not concisely share general invasion pathways in their hosts (Ivanov and Honda, 2012). Additionally, the host innate immune system has the ability to recognize and to mount tolerogenic response against commensals and inflammatory response against pathogens (Round et al., 2010; Manicassamy and Pulendran, 2011). For clarity, some studies have suggested that maggots may act selectively against pathogenic microorganisms that are found in chronic wounds and bacteria isolated directly from the larvae and their ES (Jaklic et al., 2008; Bohova et al., 2014).

The numerous properties of a given bacterium distributed in different parts of the digestive tract of *L. sericata* were responded herein in part, by evaluating the susceptibility of *P. mirabilis* isolates from different compartments of L_3_ to various antibiotics. The results not only revealed the diversity in antibiogram susceptibilities but also displayed more visibility of this pattern in the salivary glands and midgut isolates than other isolates. Thanks to extracorporeal digestion (Andersen et al., 2010), the salivary glands in the *L. sericata* are the first parts of the food canal to be in contact with the engorging environment, and the midgut is a “hot spot,” where many microbes are actively exchange their genetic materials, including antibiotic resistance genes (Dillon and Dillon, 2004; Le Roux and Blokesch, 2018). Knowledge of the sensitivity pattern of symbiotic bacteria, for example, *P. mirabilis*, may be crucial in wound healing and formulating rational antibiotic policy.

In insect populations, symbiotic bacteria could be acquired horizontally or vertically and from surrounding environments (Funkhouser and Bordenstein, 2013; Thompson et al., 2013). Association of microbiota in food sources with necrophagous flies has been considered in a few studies (Ahmad et al., 2006; Banjo et al., 2006; Förster et al., 2007; Dharne et al., 2008). In this study, six bacterial species were detected from food supplies that may come into the life cycle of *L. sericata*; however, only *E. faecalis* and *S. marcescens* kept circulation in all stages via the transstadial transmission (Table 1). Other species of bacteria may be obtained from conspecific flies. *Enterococcus faecalis* is a Gram-positive and commensal bacterium of the human/animal digestive tract (Berg, 1996; Ryan et al., 2003). It can be an opportunistic pathogen causing serious infections, namely, urinary tract infections, endocarditis, bacteremia, and wound infections (Kau et al., 2005). This lactic acid bacterium is frequently found in the small intestine of healthy humans (Rôças et al., 2004), where it chiefly survives by the fermentation of non-absorbed sugars (Murray, 1998). *Enterococcus faecalis* additionally sprang up to exploit a variety of resources by tolerating severe salt and alkalinity (Stuart et al., 2006). Similar to its eukaryotic host, *L. sericata*, this bacterium has been provided promising data for PMI estimation (Iancu et al., 2018), although its role in the MDT and biology of *L. sericata* is unclear.

In this study, *S. marcescens* were obtained from the food supplies of adult flies, live larvae (L_1_–L_3_), and the corpse of adult flies that were preserved for a long time. The bacterium is generally known to be an entomopathogen, however; it can be an opportunistic pathogen of plants, nematodes, and humans (Grimont and Grimont, 2006). Infections of *S. marcescens* have been reported in various flies, specifically apple maggot flies, *Rhagoletis pomonella* (Lauzon et al., 2013); blowflies, *L. sericata* (Meigen) (Parvez et al., 2016); fruit flies, *Drosophila melanogaster* (Miest and Bloch-Qazi, 2008); house flies, *M. domestica* (L.) (Parvez et al., 2016); stable flies, *Stomoxys calcitrans* (Castro et al., 2007); and tsetse flies, *Glossina* species (Poinar et al., 1979). It is also a well-adapted bacterium to *L. sericata* because it could survive more than 2 years in the fly’s body. In a study, the survival of ingested *S. marcescens* in house flies after electrocution was found to be up to 5 weeks (Cooke et al., 2003). The way to enter the insect host has been reported to determine the outcomes of the *S. marcescens* infections (Sanchez-Contreras and Vlisidou, 2008). It has also been indicated that the protozoan parasite, *Leishmania mexicana*, has the ability to protect sandfly host, *Lutzomyia longipalpis*, from the bacterial pathogen, *S. marcescens* (Sant’Anna et al., 2014). In this regard, we argue that the *S. marcescens* found across the *L. sericata* gut in this study is likely non-pathogenic or is supported by indigenous microbiota (bacteria with profound effects on the anatomical, physiological, and immunological development of the host) via colonization resistance. Both ideas need to be investigated in future studies.

Our knowledge of how bacteria are circulated horizontally in and vertically between the generations of *L. sericata* is limited. In general, symbiotic bacteria need such circulation to maintain their community within the host populations (Ferrari and Vavre, 2011). The results of our study found a number of bacteria e.g., *Klebsiella* species, *Lactobacillus* species, *L. garvieae*, *M. morganii*, *Providencia* species, *Pseudacidovorax intermedius*, *P. otitidis*, *V. fluvialis*, and *Ventosimonas* species that were present in two or three stages of *L. sericata*, although they had an incomplete transstadial transmission, and the sample size was insufficient to trace bacteria in further stages. Nevertheless, others such as *E. faecalis*, *M. phaeus*, *Proteus* species, *P. vermicola*, and *S. marcescens*, which were present in most of the examined stages (≤4), may have had a complete transstadial transmission. Among bacteria with the transstadial transmission, those found in adults are of particular importance for development or biological traits as the reorganization of bacteria while passing immature to adult stages occurs in pupal stage (Greenberg, 1968).

A number of 12 species of bacteria were found in eggs, five species by culture-dependent, eight species via metagenetic approach, and the species *C. freundii* by both methods. These bacteria were presumably transferred to the offspring in the ovary (transovarial) not across the eggs (transovum) because eggs were surface sterilized using immersion either in 70% ethanol or in 3% Lysol. Herein, we discuss that each transstadial and transovarial transmission route of bacteria has its relative significant for the circulation of bacteria within and between populations. This observation contradicts Singh et al.’s (2015) findings in which the transstadial transmission was more evident than transovarial transmission. The reason for this discrepancy may be due to different bacteria identification methods used and the number or type of samples examined. The number of reads and sequence lengths in the study of Singh et al. (2015) were completely different from our study. These factors may influence the identification of the bacteria at the lower levels of taxonomy and thus the inference of the horizontal or vertical circulation of the bacteria in the population of flies.

It is now widely accepted that diets and other environmental factors modulate the composition and metabolic activity of human and animal gut microbiota (Conlon and Bird, 2015; Kers et al., 2018; Ng et al., 2018; Zhang et al., 2018). The results of this study highlighted more bacterial isolates/species in specimens rearing on the sterile diets than non-sterile ones. Remarkably, two key bacteria, *P. mirabilis* and *S. marcescens*, were shared between two types of diets, which likely denote that it may be unnecessary to sterilize the eggs used in MDT. In addition, larvae specimens reared in the sterile diet did not ripen to L_3_, or very small L_3_ was generated. This result has been verified in other studies, and the immature stages of several fly species fail to develop in the substrates lack bacteria (Schmidtmann and Martin, 1992; Zurek et al., 2000).

In this study, the effect of feeding status on the bacterial load of *L. sericata* was investigated. In hematophagous insects such as sand flies, the protein-rich bolus of the blood normally causes the rapid growth of gut bacteria, and when absorption is accomplished, most bacteria were defecated with blood remains (Maleki-Ravasan et al., 2015). However, in our study, the number of bacterial isolates detected in the guts of unfed larvae was three times as large as in the fed larvae. The presence of food in the intestinal tract of larvae probably acts as a physical barrier to bacterial growth, and after the digestion and excretion process, nutrients became available to bacteria, thereby stimulating their growth. Microbial competition immediately comes to an end with the elimination of transient/invading bacteria and the regeneration of native microbiota. The species, such as *P. mirabilis*, *S. marcescens*, *E. faecalis*, *M. morganii*, and *P. urinalis*, which were found in the unfed state of the microdissected compartments, implies the resident gut bacteria. Although a more precise methodology is needed, these results indicate that the digestive process increases the number of native bacteria than other bacteria.

Among the three studied larval instars, most bacterial species were recovered from L_3_ and may be due to both the greater nutritional activity of L_3_ and the detailed study of its gut compartments. The culture-dependent method revealed the highest number of bacterial species in the salivary glands, while the metagenetic approach exposed the highest number of bacterial species in the Malpighian tubules. For the bacteria identified in both compartments, the metagenetic results were similar, and only the culture-dependent result was different, because live bacteria were detected only in the second method. These findings appear to be rational, because in the *L. sericata*, salivary glands are tissues directly contacted with the food surface bacteria, and Malpighian tubules are excretory organs where live/dead bacteria must be repelled out of the body. Likewise, the occurrence of *P. mirabilis* isolates in tracheal tubes highlights the potential role of bacteria in insect development as indicated in mosquitoes (Coon et al., 2017) and presumably in immunity through swarming motilities that suppress the growth of other bacteria. As a result, bacteria in the digestive/respiratory systems of larvae of blowflies assist in the breakdown of food and sustain the immune hemostasis, as indicated by Ivanov and Honda (2012) and Tomberlin et al. (2017).

Adult blowflies are regularly in contact with carrion (Pechal and Benbow, 2016), wounds on animals (Sanford et al., 2014), feces (Mann et al., 2015; Brodie et al., 2016), and even pollen-rich composite flowers (Brodie et al., 2015a). These resources are important for the courtship and mating behavior, obtaining nutrition required for oogenesis, or supporting the development of offspring (Tomberlin et al., 2017). Certain bacteria, including *Providencia rettgeri*, *M. morganii*, *P. vulgaris*, and *P. mirabilis*, are the initial colonizers of infested wounds, and olfactometer tests using bovine blood containing these bacteria showed that their by-products/degradation results in MVOCs that attract blowflies to colonize in those substrates. Although these bacteria had individually been attractive to the flies, their combination was reported to be more effective, and the cultures of *P. rettgeri* were found to be the most attractive ones (Eddy et al., 1975). Other results specified that MVOCs from five individual species (*K. oxytoca*, *P. mirabilis*, *P. vulgaris, P. rettgeri*, and *Providencia stuartii*) were responsible for attracting more females, resulting in more oviposition than MVOCs from *E. cloacae*, *Enterobacter sakazakii*, and *Serratia liquefaciens* (Chaudhury et al., 2010). Furthermore, the interkingdom swarming signals from a *P. mirabilis* isolated from the salivary glands of *L. sericata* and their influence on blowfly in access to the new hosts/environments have been explored carefully (Ma et al., 2012; Tomberlin et al., 2012; Liu et al., 2016). The components of MVOCs from bacterial origin, which regulate the activation responses of blowflies, have been determined as dimethyl disulfide, dimethyl trisulfide, ethanethiol, indole, isobutylamine, *p*-cresol, phenol, phenylacetic acid, phenylacetaldehyde, and skatole (Dethier, 1948; Richardson, 1966; Grabbe and Turner, 1973; Erdmann and Khalil, 1986; Chaudhury et al., 2014). However, the type of volatiles that these bacteria produce and the manner in which flies respond appear to be bacterium- or strain-specific, as indicated by Brodie et al. (2014).

In this survey, a number of 16 and 3 bacterial species identified were from the adult flies reared in the insectary and those captured from the field, respectively. *Providencia vermicola* and *Ventosimonas* species were found to be dominant bacteria in males and females, respectively. Bacteria in the genus *Providencia* are pathogens of many organisms, including humans and insects (Galac and Lazzaro, 2011). Initially, *P. vermicola* had been isolated from an entomopathogenic nematode, *Steinernema thermophilum* (Somvanshi et al., 2006); later, its pathogenic effects were approved in silkworm *Bombyx mori* (Zhang et al., 2013) and fruit fly *D. melanogaster* (Galac and Lazzaro, 2011). Moreover, it has been revealed that this bacterium is resistant to the *L. sericata* larval excreta/secreta (Jaklic et al., 2008). Recently, a member of the Gammaproteobacteria, *Ventosimonas gracilis*, has been isolated from *Cephalotes varians* ant guts, which represent a new family, genus, and species (Lin et al., 2016). In our study, the DNA of this obligate aerobic bacterium was found in female flies.

Some bacterial communities may have large influence on the life history of insects (Gurung et al., 2019). A notable instance is the necrophagous beetle, *Nicrophorus vespilloides*, in which there is a potential metabolic cooperation between the host and its microbiota for digestion, detoxification, and defense, which prolong from the beetle’s intestine to its nutritional substrates (Vogel et al., 2017). However, the direct role of the bacteria associated with *L. sericata* has not adequately been addressed in the literature either in fly ecology or in MDT process. Conversely, the effects of larval ES on bacteria related to wounds (but not bacteria isolated from non-sterile larvae) had been considered to be antibacterial, antibiofilm, and boosting antibiotics (Jaklic et al., 2008; Cazander et al., 2009, 2010a,b).

## Conclusion

The complexity of the molecules, enzymes, and AMPs involved in MDT makes it impossible to separate the wheat from the chaff and to determine the exact roles of each larval and symbiotic partner. Consequently, the first and critical step in this context is identification of bacteria present in different compartments of the *L. sericata*. Here, we reacknowledge using conventional cultivation and advance molecular techniques that *L. sericata* is associated with bacteria both in different gut compartments and different developmental stages. Various factors, including diets, feeding status, identification tool, gut compartment, and life stage, governed the bacterial species. However, the most prevalent species was Gammaproteobacterium *P. mirabilis* with different biochemical properties especially in the salivary glands and midgut isolates. Moreover, we argued that each transstadial and transovarial transmission routes of bacteria have its relative significance within and between *L. sericata* populations. Nevertheless, bacteria such as *E. faecalis*, *M. phaeus*, *Proteus* species, *P. vermicola*, and *S. marcescens* that have transstadial transmission are more important, representing the lack of adverse effect of the larval ES on these resident bacteria. The findings of this study are planned to pave the way for further research in the role of each bacterial species/strain in the insect ecology, as well as in antimicrobial, antibiofilm, anti-inflammatory, and wound healing activities.

## Data Availability Statement

The sequencing data generated for this study can be found in the NCBI under accession numbers MF399269-MF399394 for cultured and MF327011-MF327133 for uncultured bacteria.

## Ethics Statement

All phases of the study have acknowledged ethics approval from the Research Committee and Institutional Ethics Committee of the Pasteur Institute of Iran. Two diabetic foot and bed sore cases involved in this study gave their written informed consents in accordance with the Declaration of Helsinki. For the first patient, the informed consent was provided by the patient himself, but for the second one, it was written by her legal representatives.

## Author Contributions

NM-R, ND, AR, and SZ contributed to conceptualization of the project. NA and ZS performed the laboratory works. NM-R carried out the data analysis and interpretation and drafted the manuscript. NM-R and ND critically revised the manuscript. All authors read and approved the final version of the manuscript.

## Conflict of Interest

The authors declare that the research was conducted in the absence of any commercial or financial relationships that could be construed as a potential conflict of interest.

## Supplementary Material

FIGURE S1 Micro-dissected compartments of the digestive tract of third-stage larvae of *Lucilia sericata*. **(A)** foregut (showing mouth hooks, unexpanded crop, and two tubular salivary glands), **(B)** a full feed crop; **(C)** crop immediately after food discharge; **(D)** one out of four Malpighian tubules; **(E)** trachea and tracheoles.

FIGURE S2 Front and up view of a diabetic patient’s foot underwent maggot debridement therapy.

FIGURE S3 A large bed sore before and at the time of maggot debridement therapy.

FIGURE S4 Biochemical differentiation of *Shigella* species (right) and *Escherichia coli* (left) with identical *16S rRNA* gene sequences through EMB medium.

TABLE S1 Characteristics of the representative *16S rRNA* gene sequences of 21 bacterial species identified from *Lucilia sericata* using Culture-dependent method.

TABLE S2 Characteristics of the representative 16S rRNA gene sequences of 32 bacterial species identified from Lucilia sericata using Culture-independent method.
