# Supplementary figures and images for: New Insights Into Culturable and Unculturable Bacteria Across the Life History of Medicinal Maggots Lucilia sericata (Meigen) (Diptera: Calliphoridae)
Source: Front Microbiol. 2020 Apr 8;11:505. doi: 10.3389/fmicb.2020.00505 (PMC7156559; doi:10.3389/fmicb.2020.00505)

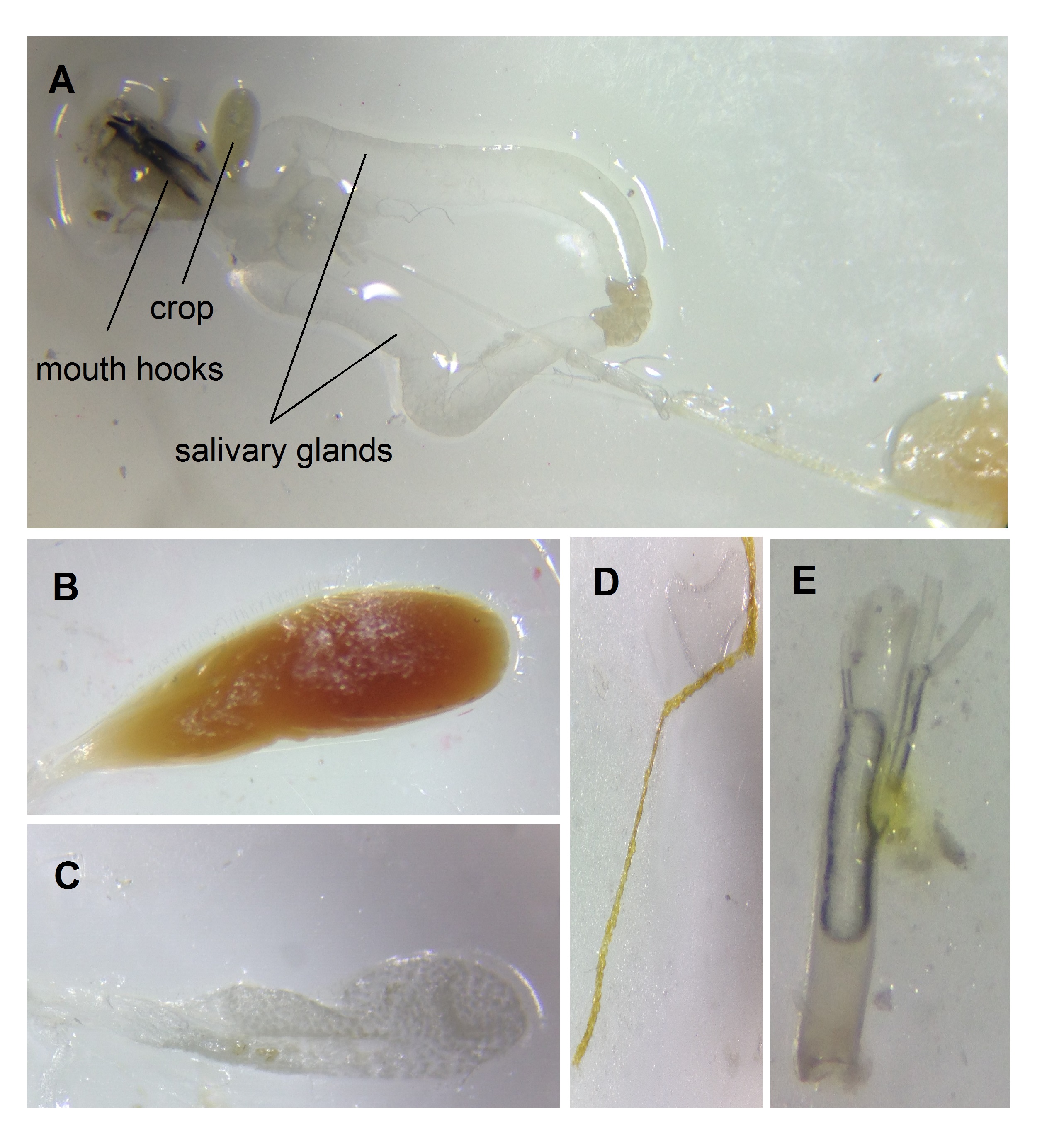

Supplement: FIGURE S1 — Micro-dissected compartments of the digestive tract of third-stage larvae of Lucilia sericata. (A) foregut (showing mouth hooks, unexpanded crop, and two tubular salivary glands), (B) a full feed crop; (C) crop immediately after food discharge; (D) one out of four Malpighian tubules; (E) trachea and tracheoles. [file Image_1.JPEG]

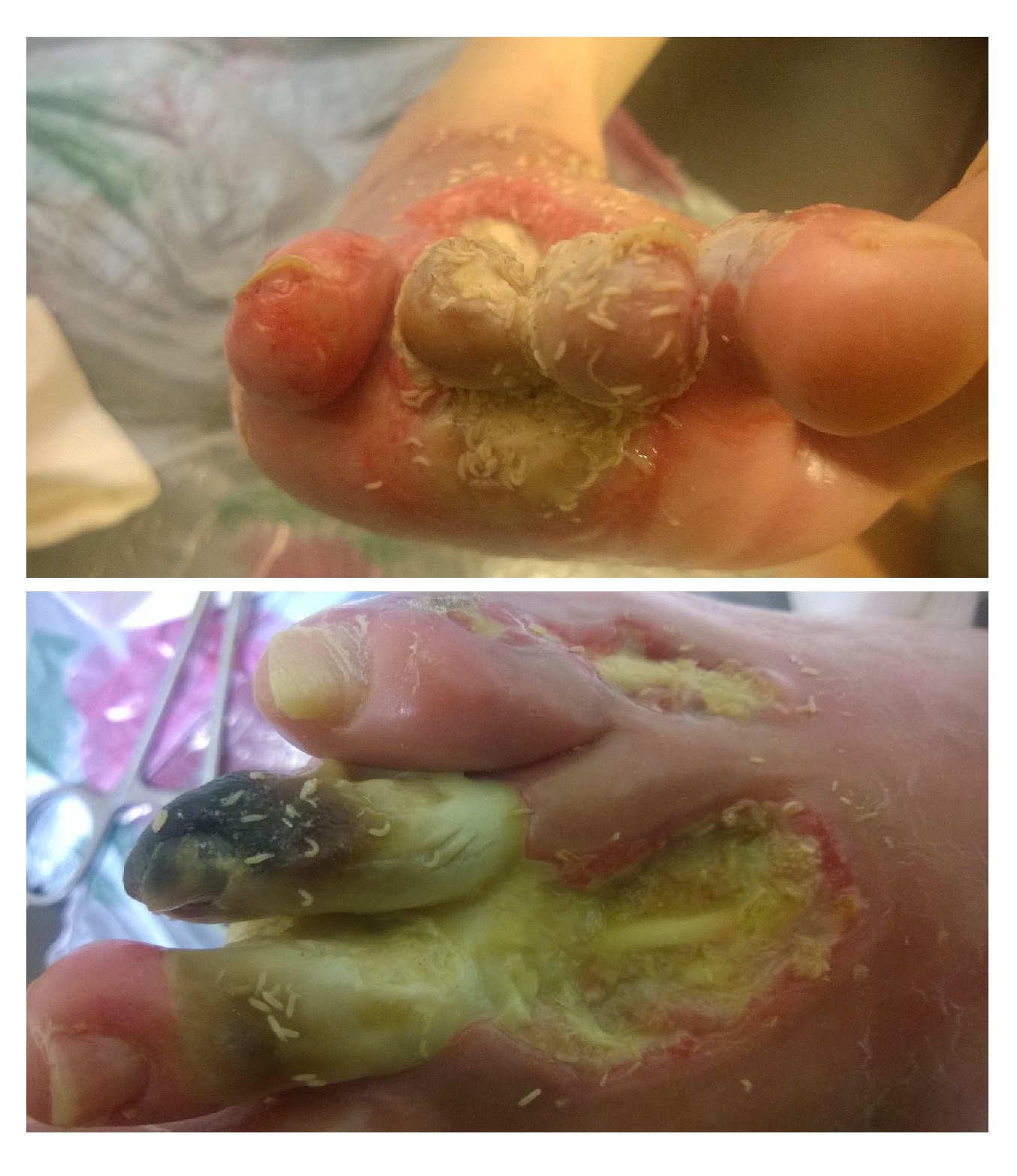

Supplement: FIGURE S2 — Front and up view of a diabetic patient’s foot underwent maggot debridement therapy. [file Image_2.JPEG]

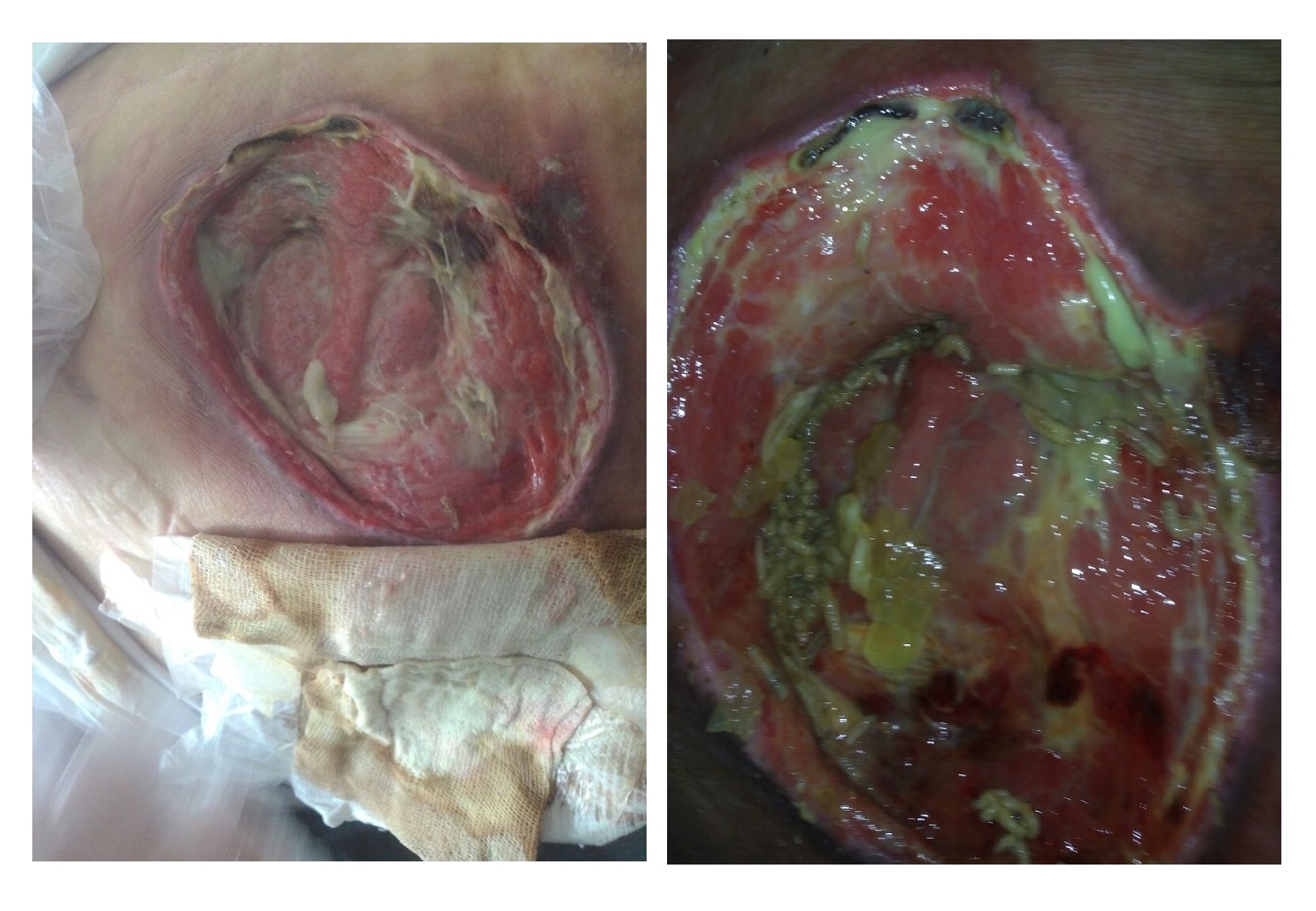

Supplement: FIGURE S3 — A large bed sore before and at the time of maggot debridement therapy. [file Image_3.JPEG]

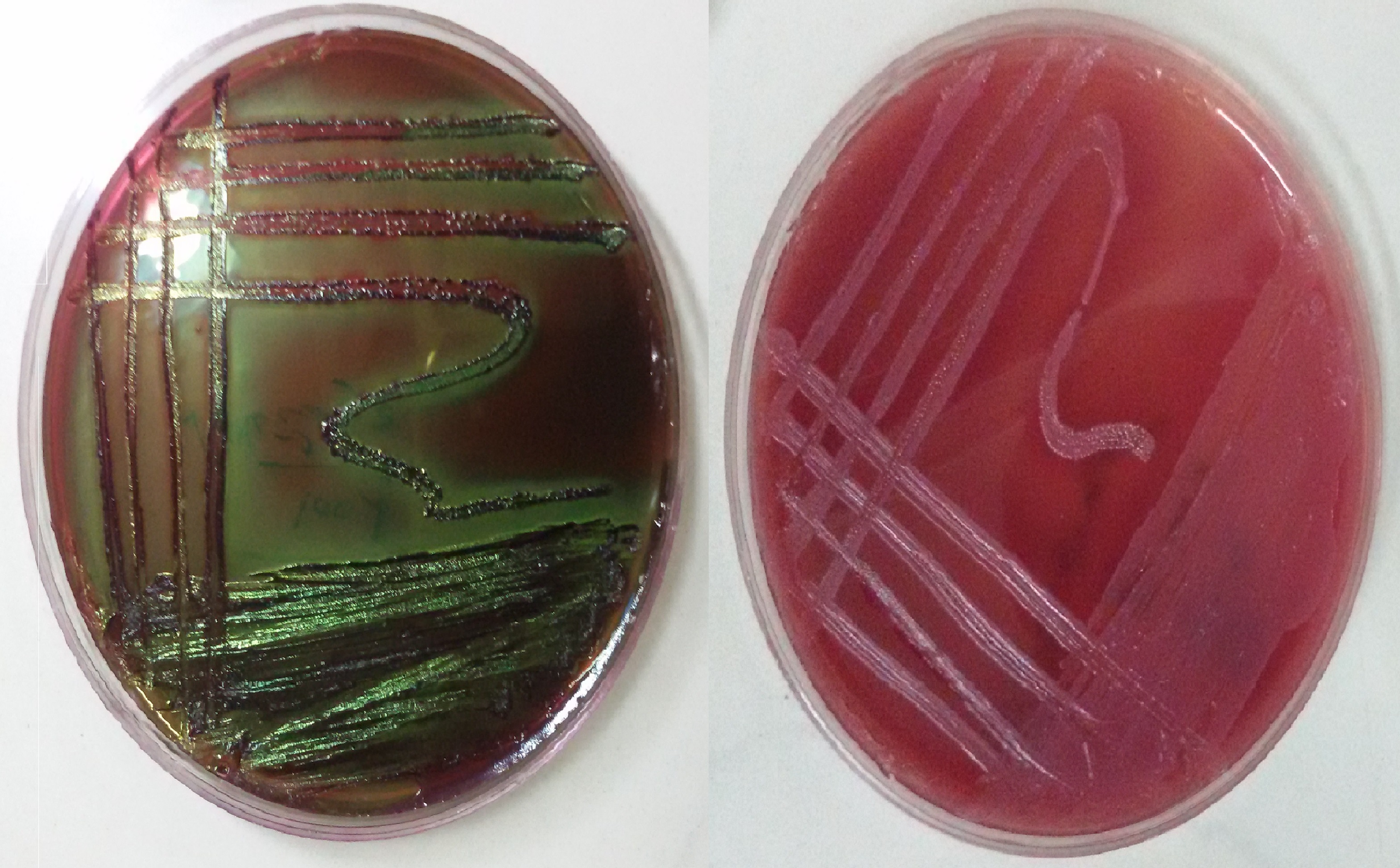

Supplement: FIGURE S4 — Biochemical differentiation of Shigella species (right) and Escherichia coli (left) with identical 16S rRNA gene sequences through EMB medium. [file Image_4.JPEG]
