# Supplementary material for: New Insights Into Culturable and Unculturable Bacteria Across the Life History of Medicinal Maggots Lucilia sericata (Meigen) (Diptera: Calliphoridae)
Source: Front Microbiol. 2020 Apr 8;11:505. doi: 10.3389/fmicb.2020.00505 (PMC7156559; doi:10.3389/fmicb.2020.00505)
Supplement: TABLE S1 — Characteristics of the representative 16S rRNA gene sequences of 21 bacterial species identified from Lucilia sericata using Culture-dependent method. [file Data_Sheet_1.pdf]

Supplementary materials

TABLE S1: Characteristics of the representative *16S rRNA* gene sequences of 21 bacterial species identified from *Lucilia sericata* using Culture-dependent method

**Assigned bacterial spp.**	**Origin**	**Length (bp)**	**Similarity %**			**Taxonomy**		**GenBank ID**
			**NCBI**	**EzBioCloud**	**leBIBI**	**Phylum**	**Family**	
Bacillus cereus	L3 retrieved from a bed sore	1369	99.86	99.86	99	Firmicutes	Bacillaceae	MF399393
Wohlfahrtiimonas chitiniclastica	L3 retrieved from a bed sore	1399	99.14	99.57	99	Proteobacteria	unclassified	MF399392
Paenibacillus urinalis	Crop of unfed L3	1444	99.04	99.12	99	Firmicutes	Paenibacillaceae	MF399389
Lactococcus garvieae	Sterile L1	1411	99.93	99.93	99	Firmicutes	Streptococcaceae	MF399387
Morganella morganii	L3 retrieved from a bed sore	1408	99.14	99.19	99	Proteobacteria	Morganellaceae	MF399386
Pseudomonas alcaligenes	Salivary glands of unfed L3	330	100	100	99	Proteobacteria	Pseudomonadaceae	MF399385
Pseudomonas japonica	Sugar meal	1404	98.99	99.13	99	Proteobacteria	Pseudomonadaceae	MF399384
Proteus mirabilis	Crop of unfed L3	1410	100	99.93	99	Proteobacteria	Morganellaceae	MF399382
Proteus vulgaris	Sterile eggs	1410	99.78	99.71	99	Proteobacteria	Morganellaceae	MF399333
Citrobacter freundii	Sterile eggs	1413	100	100	100	Proteobacteria	Enterobacteriaceae	MF399329
Providencia alcalifaciens	Non sterile eggs	1428	99.86	99.86	99	Proteobacteria	Morganellaceae	MF399328
Providencia rustigianii	Sterile L1	1429	100	99.93	99	Proteobacteria	Morganellaceae	MF399323
Providencia vermicola	Male fly from field	1410	99.78	99.78	99	Proteobacteria	Morganellaceae	MF399322
Klebsiella oxytoca	Female fly	1405	99.93	100	99	Proteobacteria	Enterobacteriaceae	MF399315
Enterococcus avium	L3 retrieved from a bed sore	1426	100	99.93	99	Firmicutes	Enterococcaceae	MF399312
Enterococcus faecalis	Sterile L1	1414	100	99.93	99	Firmicutes	Enterococcaceae	MF399311
Escherichia coli	L3 retrieved from a bed sore	1409	99.71	99.71	99	Proteobacteria	Enterobacteriaceae	MF399292
Serratia marcescens	Malpighian tubules of unfed L3	1423	100	99.64	99	Proteobacteria	Yersiniaceae	MF399279
Shigella sonnei	Sterile L1	1447	100	99.21	99	Proteobacteria	Enterobacteriaceae	MF399270
Staphylococcus hominis	non sterile pupae	1409	99.71	99.86	99	Firmicutes	Staphylococcaceae	MF399394
Myroides phaeus	Crop of fed L3	1393	97.95	97.96	98	Bacteroidetes	Flavobacteriaceae	MF399388

TABLE S2: Characteristics of the representative *16S rRNA* gene sequences of 32 bacterial species identified from *Lucilia sericata* using Culture-independent method

Assigned bacterial spp.	Origin	Length (bp)	Similarity %			Taxonomy		GenBank ID
			NCBI	EzBioCloud	leBIBI	Phylum	Family	
Serratia marcescens	Malpighian tubules of L3	768	99.35	99.35	99	Proteobacteria	Yersiniaceae	MF327132
Pseudoxanthomonas japonensis	L2	768	99.61	99.22	99	Proteobacteria	Xanthomonadaceae	MF327131
Ventosimonas sp.	Female fly	744	92	92	92	Proteobacteria	Ventosimonadaceae	MF327125
Pseudomonas sp5	Female fly	764	90	90.90	91	Proteobacteria	Pseudomonadaceae	MF327119
Pseudomonas sp4	Malpighian tubules of L3	521	99	99.42	99	Proteobacteria	Pseudomonadaceae	MF327114
Pseudomonas sp3	L2	571	100	97.72	97	Proteobacteria	Pseudomonadaceae	MF327113
Pseudomonas sp2.	Foregut of L3	762	99.87	99.87	99	Proteobacteria	Pseudomonadaceae	MF327112
Pseudomonas otitidis	Foregut of L3	762	99.87	99.87	99	Proteobacteria	Pseudomonadaceae	MF327108
Pseudacidovorax intermedius	Male fly	762	99.21	99.47	99	Proteobacteria	Comamonadaceae	MF327105
Providencia vermicola	L2	766	99.61	99.61	99	Proteobacteria	Morganellaceae	MF327103
Providencia rustigianii	L2	568	98.56	98.43	98	Proteobacteria	Morganellaceae	MF327079
Providencia rettgeri	Hindgut of L3	1499	99.61	99.61	99	Proteobacteria	Morganellaceae	MF327077
Providencia burhodogranariea	Digestive tract of L2	766	99.09	99.22	99	Proteobacteria	Morganellaceae	MF327075
Proteus vulgaris	Foregut of L3	768	98.57	98.57	99	Proteobacteria	Morganellaceae	MF327073
Proteus mirabilis	Malpighian tubules of L3	768	99.61	99.74	99	Proteobacteria	Morganellaceae	MF327052
Proteus.hauseri	Pupae	1493	99.61	99.61	99	Proteobacteria	Morganellaceae	MF327040
Klebsiella michiganensis	Foregut of L3	766	98.43	98.69	98	Proteobacteria	Enterobacteriaceae	MF327039
Morganella morganii	L2	768	98.05	98.63	98	Proteobacteria	Morganellaceae	MF327037
Citrobacter freundii	Eggs	759	98.70	98.83	99	Proteobacteria	Enterobacteriaceae	MF327034
Acinetobacter rudis	Male fly	766	95.69	95.42	96	Proteobacteria	Moraxellaceae	MF327033
Weissella koreensis	Eggs	798	99.25	99	99	Firmicutes	Leuconostocaceae	MF327032
Vagococcus fluvialis	Malpighian tubules of L3	784	97.56	97.32	98	Firmicutes	Enterococcaceae	MF327031
Lysinibacillus parviboronicapiens	Eggs	772	92.81	92.35	92	Firmicutes	Bacillaceae	MF327027
Lactococcus garvieae	Salivary glands of L3	1507	99.74	99.87	99	Firmicutes	Streptococcaceae	MF327026
Lactobacillus sakei	L2	793	100	99.87	100	Firmicutes	Lactobacillaceae	MF327025
Lactobacillus curvatus	L1	796	99.50	99.62	99	Firmicutes	Lactobacillaceae	MF327023
Clostridium perfringens	Eggs	743	99.46	99.46	99	Firmicutes	Clostridiaceae	MF327021
Bacillus safensis	Male fly	774	99.61	99.61	99	Firmicutes	Bacillaceae	MF327020
Myroides phaeus	L2	758	99.21	99.34	99	Bacteroidetes	Flavobacteriaceae	MF327019
Dysgonomonas sp.	Female fly	755	93	92.56	93	Bacteroidetes	Dysgonamonadaceae	MF327013
Propionibacterium acnes	Male fly	746	99.60	99.20	99	Actinobacteria	Propionibacteriaceae	MF327011
Chryseobacterium lactis	Malpighian tubules of L3	756	98.68	99.47	99	Bacteroidetes	Flavobacteriaceae	MF327012
